# Editorial: Imaging for lung surgery

**DOI:** 10.3389/fsurg.2022.1087973

**Published:** 2022-12-13

**Authors:** Alexander Kluge, Jean-Marc Baste

**Affiliations:** ^1^ Institute of Diagnostic and Interventional Radiology, Pius-Hospital Oldenburg, Oldenburg, Germany; ^2^ General and Cardiothoracic Surgery, University Hospital Center Rouen, Rouen, France

**Keywords:** imaging, thoracic surgery, pre-operative simulation, 3d domputed tomography, risk analysis

**Editorial on the Research Topic** Imaging for lung surgery

This research topic addresses areas of interest of thoracic surgeons, oncologists, radiologists, and computer scientists. As it emerges from the spectrum of articles collected here, the combination of their expertise is beneficial today and will become fundamental in the future. As this research topic demonstrates, CT and its morphological information are the mainstays of preoperative and intraoperative analysis and assessment, outweighing the functional information possibly contributed by MRI perfusion imaging.

The extensive scope of computational tools useful or promising for surgery is addressed by Krass et al. They give a far-reaching overview of different presently applied and emerging techniques to assess malignant lung tumors in a reliable and robust way. The relation of tumors to surrounding structures, blood supply, and potential resection margins is of paramount importance to surgeons. Thus, risk assessment for general operability, resection strategy, and surgical planning and guidance are profoundly affected by computer assistance and imaging. Remarkable commonalities with liver surgery planning in some areas of lung surgery planning are outlined. Future developments may most likely see increasing use of deep learning algorithms for pre-operative and intraoperative decision-making.

One field of immense practical relevance is the identification of segmental planes for the resection of small pulmonary nodules. This is performed preoperatively. A systematic review and meta-analysis of the literature performed by Xiang et al. assessed the impact of preoperative three-dimensional lung simulation on intraoperative outcomes compared to non-3D-procedures. In nine selected studies included in this review, parameters such as conversion rate, operative time, and postoperative indicators (postoperative hospital stay, total number of complications) as well as postoperative complications were searched for. Most notably, all of the aforementioned parameters significantly favored preoperative 3D simulation and planning. The authors conclude that preoperative 3D lung simulation improves surgical accuracy and safety and may be worthy of clinical promotion. Totally agree and well demonstrated. The 3D planning for sublobar resection, especially segmentectomies, should become the gold standard in the near future.

While retrospective analysis and monocenter studies evaluate the usefulness of 3D-computed tomography visualization and simulation exist, a prospective randomized trial is lacking. This void is addressed by Niu et al. They present an ongoing prospective randomized multicenter trial (DRIVATS) to verify the usefulness of preoperative 3D reconstruction CT compared with standard chest CT in segmentectomy. If successfully completed, this multicenter prospective study will provide a higher level of evidence for the use of 3D reconstruction CT in segmentectomy.

Zhang et al. assessed techniques applied to intraoperatively assess the intersegmental plane in order to allow for segment resection. Historically, inflation/deflation techniques have been performed, followed by jet injection techniques and different staining techniques to visualize the intersegmental plane. Instead of determining and visualizing the target area, a depiction of the area to be preserved proved to be beneficial and feasible. Future developments will most likely see the central role of 3D simulation, and even intraoperative guidance by virtual reality in video-assisted thoracoscopic lung surgery, which may become even better with the robotic platform.

Two case reports round off this research topic. A case described by Sicolo et al. highlights the prerequisite of an interdisciplinary approach to complex and unusual cases when operability can be assessed and assured only after careful evaluation and treatment, in this case by interventional radiology.

The second case report by Xing et al. depicts the “smoking gun” of a very rare complication in the making. Cerebral air embolism after percutaneous thoracic intervention is exceedingly rare but potentially disastrous for the patient. Thus this very well-documented case is a most welcome heads-up for all of us working in the field.

To conclude, we thank all the authors and reviewers for their work and their valuable contributions to this research topic. The publications gathered here will advance future developments with ever further integration of imaging into thoracic surgery.

